# Home Virtual Visits for Outpatient Follow-Up Stroke Care: Cross-Sectional Study

**DOI:** 10.2196/13734

**Published:** 2019-10-07

**Authors:** Ramana Appireddy, Sana Khan, Chad Leaver, Cally Martin, Albert Jin, Bryce A Durafourt, Stephen L Archer

**Affiliations:** 1 Division of Neurology Department of Medicine Kingston Health Sciences Centre Kingston, ON Canada; 2 Canada Health Infoway Toronto, ON Canada; 3 Stroke Network of Southeastern Ontario Kingston Health Sciences Centre Kingston, ON Canada; 4 Department of Medicine Kingston Health Sciences Centre Kingston, ON Canada

**Keywords:** telemedicine, eHealth, eVisit, mobile health, health services accessibility

## Abstract

**Background:**

Timely, in-person access to health care is a challenge for people living with conditions such as stroke that result in frailty, loss of independence, restrictions in driving and mobility, and physical and cognitive decline. In Southeastern Ontario, access is further complicated by rurality and the long travel distances to visit physician clinics. There is a need to make health care more accessible and convenient. Home virtual visits (electronic visits, eVisits) can conveniently connect physicians to patients. Physicians use a secure personal videoconferencing tool to connect to patients in their homes. Patients use their device of choice (smartphone, tablet, laptop, or desktop) for the visit.

**Objective:**

This study aimed to assess the feasibility and logistics of implementing eVisits in a stroke prevention clinic for seniors.

**Methods:**

A 6-month eVisit pilot study was initiated in the Kingston Health Sciences Centre stroke prevention clinic in August 2018. eVisits were used only for follow-up patient encounters. An integrated evaluation was used to test the impact of the program on clinic workflow and patient satisfaction. Patient satisfaction was evaluated by telephone interviews, using a brief questionnaire. Access and patient satisfaction metrics were compared with concurrent standard of care (patients’ prior personal experience with in-person visits). Values are presented as median (interquartile range).

**Results:**

There were 75 subjects in the pilot. The patients were aged 65 (56-73.5) years, and 39% (29/75) resided in rural areas. There was a shorter wait for an appointment by eVisit versus in-person (mean 59.98 [SD 48.36] days vs mean 78.36 [SD 50.54] days; *P*<.001). The eVisit was also shorter, taking on an average of only 10 min to deliver follow-up care with a high degree of patient satisfaction versus 90 (60-112) min for in-person care. The total time saved by patients per eVisit was 80 (50-102) min, 44 (21-69) min of which was travel time. Travel distance avoided by the patients was 30.1 km (11.2-82.2). The estimated total out-of-pocket cost savings for patients per eVisit was Can $52.83 (31.26-94.53). The estimated savings (opportunity cost for in-person outpatient care) for our eVisit pilot project was Can $23,832-$28,584. The patient satisfaction with eVisits was very good compared with their prior personal experience with in-person outpatient care.

**Conclusions:**

The eVisit program was well received by patients, deemed to be safe by physicians, and avoided unnecessary patient travel and expense. It also has the potential to reduce health care costs. We plan to scale the project within the department and the institution.

## Introduction

### Barriers to Care

Canadians face many barriers while accessing outpatient health care services, including accessibility, availability, acceptability, and personal choice [1,2]. In a recent survey of the western Canadian provinces, 10% of adults with chronic health conditions reported having barriers to accessing outpatient primary care [2]. The overall health care experience was found to be poor in patients with chronic health conditions even when they reported good access because of a perceived failure of the system to meet their needs [3]. Similar barriers to outpatient specialist care exist and particularly impact seniors (aged 65 years and older) because of their higher prevalence of chronic health conditions and frailty. Seniors are also more likely to have reduced functional capacity, lower socioeconomic status, reduced independence, cognitive decline, and driving restrictions [4-6]. Patient-centered care has become a critical component of health policy worldwide and is best summarized by the Picker’s principles of patient-centered care [7]. To optimize health system performance, the Institute for Healthcare Improvement developed the triple aim framework, the goals of which are to improve the patient care experience (including quality and satisfaction), improve the health of populations, and reduce the per capita cost of health care [8]. Evidence suggests that health system transformation needs to be reformed from a patient-centric perspective to meet the health care needs of seniors [4,9,10].

### Telemedicine and Virtual Visits

Traditional practice utilizes in-person interaction to establish the patient-physician relationship and to complete a comprehensive clinical evaluation, including history and physical examination. However, follow-up care, including symptom management, diagnostics, and therapeutic decision making require less in-person interaction and may be achieved by virtual visits. Virtual visits, also known as eVisits, are a secure, 2-way digital communication between health providers and their patients. eVisits may include emails, short message service text messaging, and videoconferencing [11]. A recent study from British Columbia suggests that virtual visits in primary care are associated with a high degree of patient satisfaction and positive system outcomes [12]. There is patient demand for electronic health services. In a 2018 national survey, only 6% of Canadians over the age of 16 years said that they could currently visit their health care provider online by video, whereas among those who could not, 47% desired such access [13]. Health care professionals also perceive that offering health care that is convenient to the patient is an essential aspect of good clinical medicine [14]. There is also a growing call for virtualization of health care by health care professionals, policy makers, and industry leaders [14-16].

eVisits and other telemedicine modalities across Ontario, Canada, are facilitated by technologies provided by the Ontario Telemedicine Network (OTN), a not-for-profit organization funded by the Ontario Ministry of Health and Long-Term Care (MOHLTC). For the purposes of this publication, “eVisit” refers to personal, secure videoconferencing between the health care provider and the patient. Unlike conventional telehealth modalities, an eVisit does not need new infrastructure, such as dedicated videoconferencing equipment, peripheral devices, or a telemedicine facility, and the patient remains in their home. In an eVisit, the physician and patient interface using electronic equipment that is widely available, such as smartphones and tablets. The traditional telemedicine model with the patient at a remote site (satellite site) reduces the patient travel burden but is still costly to the health care system as significant infrastructure is used at both ends. eVisits have been shown to be feasible, acceptable, and yield similar clinical outcomes compared with in-person patient cohorts in an interdisciplinary obesity treatment program for adolescents in Ontario, Canada [17].

Whether eVisits would also be beneficial to seniors, a group traditionally viewed as being less technologically adept, was tested in a 6-month pilot project in a high-volume stroke prevention clinic in Ontario at the Kingston Health Sciences Centre (KHSC). The results of this pilot program indicated that the eVisit was well received by patients and has the potential to provide cost savings to both patients and the health care system.

## Methods

### Study Setting

**Figure 1:**
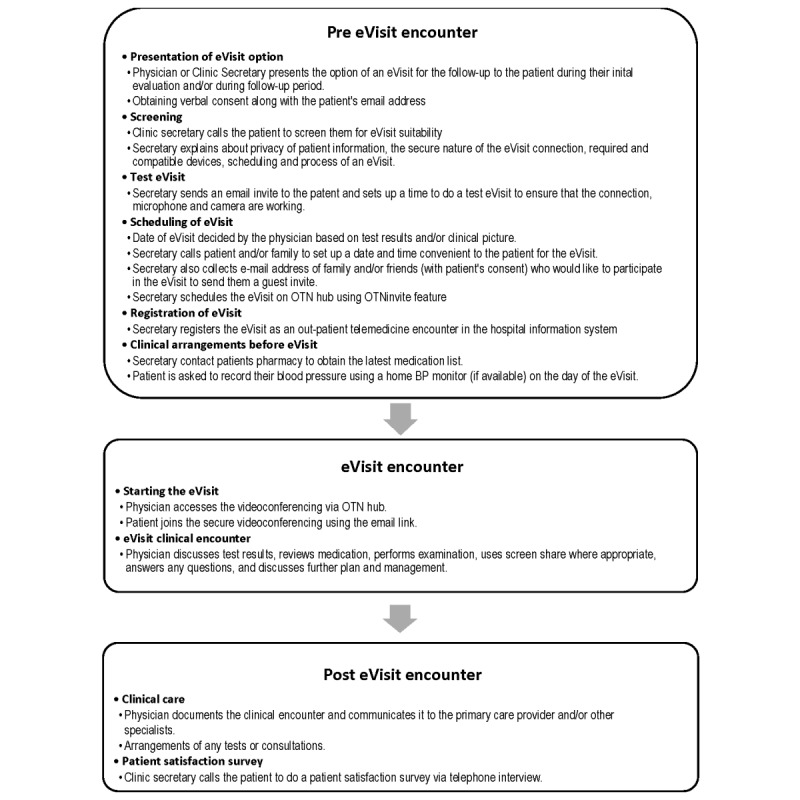
Electronic visit workflow. BP: blood pressure; OTN: Ontario Telemedicine Network.

The eVisit pilot study was initiated at KHSC in August 2018 for a 6-month period with the objective to assess the feasibility and logistics of implementing eVisits in an adult specialty disease clinic catering predominantly to seniors. An integrated evaluation was designed to test the impact of the pilot program both on clinic workflow and patient satisfaction using a telephone survey. The workflow of the eVisit intervention in the stroke prevention clinic is presented in Figure 1. The selection criteria used for an eVisit are presented in Textboxes 1 and 2. The eVisit was done through a secure Web platform hosted by OTN. The physician used a desktop computer equipped with a Web camera and a microphone for all the eVisit encounters. The patients used the device of their preference. In addition, the eVisit platforms offer other features including a guest invitation option, wherein up to 6 more participants (family, friends, or other health care team members) can join in the videoconference.

**Inclusion criteria for patients suitable for an independent electronic visit: patient characteristics.**

- No cognitive issues.
- No loss of communication abilities.
- No physical deficits and loss of functional abilities.
- No sensory or perceptual deficits.
- No visual field deficits with functional implications.

**Inclusion criteria for patients suitable for an independent electronic visit: electronic visit technical eligibility.**

- Patient/substitute decision maker (SDM) willing to do an electronic visit (eVisit) for follow-up care.
- Patient/SDM has internet-enabled device (smartphone, tablet, or computer).
- Patient/SDM has access to an internet connection.
- Patient/SDM has a secure place to perform an eVisit.

The study subjects were selected from patients routinely seen in the stroke prevention clinic using prespecified criteria (Textboxes 1 and 2). This was used only for follow-up appointments, and the characteristics of the patients, clinical or eVisit characteristics, patient satisfaction survey, and the impact on the clinic wait times are described. The patient satisfaction survey asked the patients to report their experience with eVisits compared with their personal experience with prior in-person health care; thus, the subjects acted as their own controls. Follow-up wait times for patients seen by an eVisit were compared with a control arm consisting of patients seen for in-person follow-up in the stroke prevention clinic over the same time period. The follow-up wait time is measured as the time from initial evaluation to follow-up appointment.

eVisits were used only for the follow-up visits. Patients had to fulfill the *patient characteristics* and *eVisit technical eligibility* criteria as shown in Textboxes 1 and 2. Individualized decisions were made for patients who were willing but did *not* meet *patient characteristics* eligibility *and* had a family member or substitute decision maker that fulfilled the eVisit technical eligibility.

The definition for *senior* status used for this study is *aged 65 and over* as adapted from Statistics Canada [6]. The rural residence is defined based on the second character of the 6-digit postal code of the patient’s home address [18]. Savings on patient time (total time, travel time, and in-person visit time) and travel distance avoided were estimated using these definitions (Multimedia Appendix 1). The in-person visit time was conservatively estimated at 30 min in addition to the travel time.

### Methodology for Economic Analysis

We also performed a preliminary economic analysis to estimate hypothetical out-of-pocket (OOP) patient cost savings of eVisits and opportunity costs of in-person outpatient care. Opportunity cost are defined as benefits foregone by the particular use of resources, resources which could be otherwise allocated for other health care priorities [19]. These are hypothetical estimates based on some assumptions mentioned below and not based on actual economic data (income, employment status, outpatient costing, etc) from the patients in the pilot or the hospital.

#### Out-of-Pocket Cost Estimate

The OOP expenses were not captured using a specially designed survey; however, approximate reasonable OOP expenses were estimated using the cost of travel, parking, potential loss of pay, and total cost (Multimedia Appendix 1). As there are no current data on the impact of virtual care on OOP expenses for outpatient care, we attempted to estimate this in our study. The potential loss of pay for an adult obtaining in-person care is estimated using a hypothetical estimate of what an adult Canadian older than 25 years working full-time would lose on average if they had visited a doctor for an in-person visit, assuming the travel distance and time spent per in-person visit are similar to the study cohort [20]. The loss of pay was estimated using the average full-time hourly wage of Can $28.98 per hour for Canadian adults aged 25 years and older based on 2018 Statistics Canada data [20]. Additional OOP expenses such as food expenses, childcare costs, loss of pay for a family member, or other caregiver costs were not considered for our analysis of OOP estimates.

#### Outpatient Hospital Cost and Opportunity Cost of In-Person Outpatient Hospital Care

Our institution does not collect or report outpatient costing to Canadian Institute for Health Information (CIHI), so we used the available provincial outpatient costing data for reference [21-23]. The outpatient or ambulatory care costs are reported only by a few hospitals across the country and include the direct costs (nursing, diagnostic tests, operating, and recovery room), functional center indirect costs (meals, facilities management, and plant operation), and costs for patient-specific drugs and supplies. Using the Comprehensive Ambulatory Classification System (CACS) developed by CIHI for 2 codes (E751: General Signs, Symptoms, Examinations and Investigations; E752: Other Medical and Follow-up Care), the outpatient hospital costs in Alberta (interactive health data application) and Ontario (Ontario Case Costing Initiative Costing Analysis Tool) for 2016/17 were used as reference for estimating the outpatient hospital costs for our eVisit cohort [21-23].

### Statistical Analysis

Data were entered into Epidata software (The Epidata Association, Denmark) and were analyzed using STATA v15.0 (StataCorp LLC, USA). The data was analyzed using summary statistics and Wilcoxon Signed Rank Sum test for paired data. Values are stated as the mean and interquartile range (IQR). A *P*<.05 is considered statistically significant.

### Ethics Approval

Ethics approval for the pilot study was obtained, permitting for chart review and data collection (Queen’s University Ethics ROMEO # 6025439).

## Results

### Baseline Data

There were a total of 75 eVisits from August 2018 to January 2019. The details on the overall clinic volumes and the number of eVisits are provided in the Multimedia Appendix 2. A formal screening log was not maintained; however, some of the factors that influenced the patient uptake include lack of interest, lack of technology, as well as physician engagement in offering eVisits. Promotion of the eVisit by the physician resulted in higher uptake compared with engagement by the clinic secretary. During the pilot project, 40.2% (76/189) of the follow-up visits were through eVisit.

The mean (SD) and median (IQR) age of the patients was 63.7(14.3) and 65 years (56-73.5), respectively. Of the study patients, 67% (50/75) were male, 51% (38/75) were under age 65, 32% (24/75) were aged 65-75, and 17% (13/75) were over age 75. Mobile internet devices, including tablets (68%; 51/75) and smartphones (24%; 18/75), were most widely used for the eVisits, likely because of ease of use and setup. Laptops were used for 7% (5/75) and desktops were used for 1% (1/75) of eVisits. The mean (SD) and median (IQR) time spent by the physician and the patient for an eVisit encounter was 9.81 (4.46) and 10 (9-12) min, respectively. The proportion of rural residents who performed eVisits was 39% (29/75). A single family member accompanied the patient in 60% (45/75) of the eVisit encounters. A total of 11% (8/75) of the patients were at their place of work for the eVisit, the eVisit was done in a secure location selected by the patient, and none of them needed to take time off work.

The Wilcoxon signed-rank test showed significant reduction (*P*<.001) in the mean (SD) wait times for follow-up for in-person (mean 78.36 days, SD 50.54 days) compared with eVisit follow-up (mean 59.98 days, SD 48.36 days).

The savings on travel avoided, time savings, and direct patient OOP expenses are presented in Multimedia Appendix 3. The median value for total time saved and total travel distance avoided are 80 (50-102) min and 30.1 (11.2-82.2) km, respectively.

The various components of patient care during the eVisit included, when relevant, a review of imaging tests (33%; 24/72), cardiac tests (43%; 31/72), lab tests (26%; 19/72), consults from other specialists (28%; 20/72), medication reconciliation (93%; 67/72), and potential new tests or interventions (50%; 36/72). Screen sharing was used for 28% (20/72) of eVisits. The diagnosis at the time of eVisit included stroke (49%; 35/72), transient ischemic attack (33%; 24/72), migraine (3%; 2/72), epilepsy (8%; 6/72), or other (7%; 5/72).

A telephone patient experience survey was also completed by patients that had an eVisit with a good survey response (46%; 33/72). The degree of patient satisfaction captured using the survey questionnaire was very high (Table 1). Almost all of the respondents agreed that the eVisits saved them time (100%;33/33), money (97%;32/33), and avoided traveling to the doctor’s office (100%;33/33). All the patients who responded reported having a better experience via eVisit compared with an in-person visit and felt that their health issue was appropriately addressed during the eVisit. All the patients that had an eVisit were very willing to use the eVisit for further follow-up encounters. More than 90% (31/33) of the patients reported that they would strongly recommend an eVisit option or process to their friends and family. This was the first virtual health care encounter for all the patients that were involved in the eVisit pilot program. All were pleased with the convenience it offered and shared their experiences on a voluntary basis and were not enrolled in a formal qualitative study (Textbox 3).

**Table 1:** Patient experience from electronic visits (N=33).

Question		n (%)
**Did eVisit^a^** **save you time?**		
	Yes	33 (100)
**Did eVisit save you money?**		
	Yes	32 (97)
**Did eVisit allow to avoid traveling to your doctor or specialist?**		
	Yes	33 (100)
**Do you think if your health issue was addressed appropriately during the eVisit?**		
	Yes	33 (100)
**Did you feel that the security and privacy of your health care information were protected during the eVisit?**		
	Yes	33 (100)
**How is the experience of care from using the eVisit compared with an in-person encounter?**		
	Better	12 (36)
	Same	19 (58)
	Not sure	2 (6)
	Worse	0 (0)
**Would you use eVisit again?**		
	Definitely	31 (94)
	Probably	2 (6)
	Neutral, probably not, definitely not, not sure	0 (0)
**How likely are you to recommend the eVisit to a friend on a scale of 0-10?**		
	0-7	2 (6)
	8-100	31 (94)

^a^eVisit: electronic visit.

### Economic Analysis

The estimates for OOP costs saved are presented in Table 2. The median estimate for total OOP patient cost savings by using the eVisit instead of the in-person visit was Can $52.83 (31.26-94.53).

The outpatient hospital-based health care costs in Ontario and Alberta for codes E751 and E752 based on CIHI CACS for 2016/17 are provided in the Multimedia Appendix 4 [21-23]. Using the Ontario data as the reference, the estimated cost of outpatient hospital care for our eVisit cohort was between Can $23,832 to 28,584.

**Excerpts of written feedback received from patients and family about the electronic visit experience.**

“It is really important for a patient to have a proper conversation with their doctor, to ask questions, and to get answers which put my mind at rest. I felt as if I was in your office talking to you face to face. Not having to arrange transportation to get to your office was a real help. Now that I am not able to drive, mobility within the community is a real issue. I hope that this will be something you can offer to patients regularly.” [Female patient, aged 90 years]

“The e-visit saved time and a lot of stress that is involved in taking an elderly patient out especially in bad weather.” [Family of a male patient aged 88 years]

“It was nice not to have had to drive to the hospital, pay for parking, and make the physical effort of getting to the appointment destination.” [Male patient, aged 88 years]

**Table 2.** Estimated out-of-pocket cost savings to patients in Canada.

Out-of-pocket expenses category	Total (Can $)	Can $, mean (SD)	Can $, median (interquartile range)
Patient self-reported cost for in-person visit (n=24)	417	13.4 (14.5)	10 (5-15)
Estimated travel cost for patients	2384.46	33.13 (36.92)	16.55 (6.16-45.21)
Estimated total out-of-pocket savings	5393.97	74.92 (57.99)	52.83 (31.26-94.53)

## Discussion

### Electronic Visit Implementation

We demonstrated that eVisits could be successfully implemented for secondary prevention of stroke in an adult neurology clinic catering predominantly to seniors. eVisits are time-efficient for physicians and patients, taking a median time of 10 min while avoiding the logistical challenges of an in-person encounter and reducing OOP expenses. Patient satisfaction is very high with the eVisits. A significant proportion (33%;11/33) of our cohort reported the experience to be better than an in-person encounter. During the eVisit, it is possible to perform most of the conventional components of clinical care that happen during a routine follow-up clinic visit for this patient population. The proportion of cancellations and no-shows is minimal, highlighting the impact of the eVisits on the overall efficiency of this model of ambulatory care. There is a significant reduction in the wait times for the patients via eVisit compared with in-person follow-up, which is likely because of the lack of the need for traditional health care infrastructure. The direct translatable savings to the patients with regard to OOP expenses for travel avoided and time saved are substantial.

### Conventional Telemedicine Versus Electronic Visits

There is extensive literature describing the positive impact of conventional telemedicine modalities on access to health care globally [24-28]. Conventional telemedicine modalities such as remote videoconferencing between a host site and peripheral telemedicine satellite site have reduced the need for patient travel, reduced wait times, and improved coverage. However, these conventional models still require the patient to go to the satellite telemedicine site and are expensive, requiring significant specialized infrastructure and personnel at the satellite site. eVisits refine and simplify telehealth (for appropriate applications) by keeping the patient at home, reducing the need for any travel, and eliminating the need for specialized health care infrastructure.

### Use of Virtual Visits

The use of eVisits has grown in the last few years across North America [29]. Although this model is embraced by patients, physicians, insurance providers, and policy makers, the virtual care model is used for a few conditions in primary care [14,15,30,31] but is not broadly utilized by Canadian family physicians [32]. Broadly, there are 2 models of eVisit (virtual visit) currently available in Canada and the United States—a pay per use model and an insured model. In a pay per use model, also referred to as Direct to Consumer, the consumer (patient and/or family) pays a fee to access a physician for a health care consultation through a virtual visit [33]. In the insured model, virtual visits are covered by private or public health insurance. They are predominantly used in primary care with the majority of the use restricted to routine or common primary health conditions or situations including the common cold, skin rash, and prescription renewals [12]. Published reports of eVisits (home virtual visits) is limited to certain specific diseases or conditions such as acute respiratory illnesses or urinary tract symptoms [34-38]. Overall, specialist use of virtual visits is higher (9%) compared with primary care (4%) [32]. The use of virtual visits for specialist care is limited in Canada, with the predominant use in psychiatry (personal communication with OTN). Thus, our experience is one of the first reports on the use of eVisits by specialists in Canada.

### Electronic Visits Address Triple Aim and Patient-Centered Care

#### Patient Experience of Care

eVisits were associated with high patient satisfaction when employed in primary care settings [12]. The KHSC pilot demonstrated that the eVisits offer a high degree of patient satisfaction in specialty care among seniors and align with Picker’s principles of patient-centered care [7]. In our study, seniors (aged 65 and older) constituted 49% (37/75) of the eVisit clientage, suggesting an increased acceptance of virtual care in this group. The results from the patient experience survey suggest that the eVisit process positively addressed patients’ perceptions of accessibility and acceptability. The impression that an eVisit is better than an in-person encounter reported by 33% (11/33) of stroke patients in our pilot program is likely because of ease of facilitating an in-home follow-up consultation, avoiding the time, effort, or stress of arranging transportation, travel, parking, waiting in the clinic, loss of pay, need for caregiver assistance, and associated time savings. Another major advantage of the eVisits is avoiding the significant influence of weather and road conditions on commuting in the winter. The patient also has the flexibility of scheduling their follow-up eVisit at a time and location convenient for them and their family. Family members also experience a benefit by being able to join the eVisit remotely, offering increased support to patients, which is particularly crucial for seniors. Another value to the eVisit context was the ability to complete an accurate medication reconciliation as patients always had access to their actual medications at home, and they could show the real prescription with labels, and these were tallied with the medication list obtained from the patient’s pharmacy before the eVisit.

#### Population Health

Perhaps one of the most significant outcomes we report is the reduced patient wait time-to follow-up. eVisits allowed the physician to see patients sooner than would be possible for an in-person encounter, thus increasing the availability of health care. The technology supporting eVisits also provided the ability to share imaging or echocardiographic findings with the patient in addition to sharing medical illustrations, enabling and facilitating patient education, understanding, and empowerment. eVisits also offer the ability to identify risks and patient vulnerabilities sooner, improve treatment adherence, and support behavioral and care interventions to improve speech, mobility, and enhance access to home care or community-based care or allied health services. Flexible scheduling allows physicians to be more productive with their time, enabling them to distribute their clinical activity to accommodate other commitments including teaching, research, and administration. In addition to increased productivity, using eVisit has the potential to address some of the significant contributors to physician burnout (including work and organizational factors), which can in turn have consequences on patient care and health care costs [39].

#### Reducing Per Capita Cost

##### Reducing Out-of-Pocket Patient Cost

The eVisit offers the potential for a significant reduction in per capita costs for outpatient care. The estimated direct OOP cost savings for a single in-person visit is considerable. This could be much higher if accounting for multiple health care encounters. Our estimated OOP cost saving per visit is probably conservative; real savings would vary significantly based on the individual’s hourly wage, employment status, other personal factors, and visit characteristics.

##### Opportunity Cost to Health Care

Opportunity cost refers to the cost or money that the health care system could have allocated or used for similar or different interventions [19]. The estimates of the opportunity cost of using in-person care for our study cohort are Can $23,832 to $28,584. The opportunity cost could represent an opportunity to provide outpatient in-person health care to a different segment of the population. Thus, with the same health care budget, health care services could be offered to a higher number of patients, driving down per capita health care expenditure. Alternatively, this opportunity cost could be redirected to other high priority areas to increase health care outcomes and efficiency. Scaling of the project within health care organizations could have a significant impact.

### Limitations of the Electronic Visit

Some of the disadvantages of the eVisits relate to the technology itself. The service cannot be offered to patients who do not have an internet enabled device and/or sufficient internet connection speed, thus potentially limiting the access to home-based eVisits to patients with lower socioeconomic status. In addition, internet access and speed are limited in some geographic areas, especially in rural and remote communities. Moreover, patients with physical, cognitive, and language disabilities may find it hard to use the technology or navigate the appropriate software on their own. Another disadvantage is the inability to do an in-person clinical examination, limiting the utility of the eVisits in some clinical scenarios. However, the video-based examination has been shown to be reliable and valid [40,41].

### Strengths of This Project

The strengths of the pilot study include implementation of a successful eVisit program for outpatient follow-up in a specialty stroke clinic catering largely to a senior population. The combination of high degree of patient satisfaction with potential savings of both time and money holds promise for economically improving access to care. The mean time spent per eVisit, including the documentation of the clinical encounter, was 10 min, which is comparable with the time allocated for the physician-patient portion of a typical in-person clinical contact. We believe that our choice to perform a test eVisit before the physician-patient visit reduced the chance of communication technical difficulty and resulted in very successful physician eVisits with 5% (4/81) failure rate and 3% (2/81) no-show rate. This pilot study will inform the expansion of the eVisit project to other specialty clinics within the organization as the next phase.

### Limitations of This Study

Some limitations of the study include the limited sample size and pilot duration. The scope of the project was narrow, involving 1 specialty clinic. These limitations prevent broader generalizability. There is a potential for bias in assessing patient satisfaction as a result of using a brief telephone survey with nonprobability sampling. The economic analysis of outpatient costs, as well as opportunity cost for in-person care, needs to be corroborated in future studies across multiple organizations. The outpatient health care costing data may vary amongst different health care organizations.

### Future Directions

The limited uptake of virtual care services such as eVisits by physicians was recently reported in a 2018 survey, highlighting the need for appropriate reimbursement or alternative payment models as well as improved technology, privacy and security guidelines, and support from clinician associations and governance bodies [32]. Physician eVisits have recently been approved for reimbursement by the MOHLTC, Ontario, as part of a pilot project through OTN, which may increase utilization. However, technology integration and improvements, workflow, and processes or quality improvements, and privacy and security guidelines are required to integrate eVisits into the health care system seamlessly. In our pilot study, the majority of patients used a tablet or mobile phone, likely because of the widespread ownership of such devices and ease of use with internet-enabled smart devices even among seniors in Canada. This raises the potential to develop and integrate more mobile-based telehealth solutions. On the basis of the acceptance and efficacy of the pilot study, there has been a 40.2% (76/189) conversion of follow-ups from in-person to eVisit in the stroke prevention clinic pilot catering to seniors.

To address some of the limitations of eVisit, *eVisit stations* could be established at community health care centers, where local staff could be easily trained to help set up an eVisit. This could allow patients living in rural or remote areas, without access to the internet or who are unable to independently use the eVisit system, to attend an appointment locally and avoid lengthy travel to an urban center. A more comprehensive health economic analysis of eVisits for outpatient care is warranted in any future studies given the potential for reduction in per-capita health care costs. In addition, the impact of the eVisits on patient outcomes, wait times, readmission rates, and impact on the use of urgent care or emergency departments should be evaluated.

### Conclusions

eVisits were implemented successfully for an outpatient follow-up clinic for adult stroke patients in our pilot study. eVisits were well received by patients and consistent with a patient-centered care philosophy. eVisits have the potential to significantly transform the ambulatory clinic practice by addressing some of the barriers to care and improving patient experience, reducing per capita health care costs, and improving population health. Such a transformative change needs the involvement of health care professionals, health services researchers or economists, hospital leadership, clinician associations, and health system governance bodies at the regional and provincial levels to inform evidence-based practice guidelines and sustainable models of care. eVisits are scalable and could be expanded to additional specialty programs, a move that is underway at KHSC.

## Appendix

Multimedia Appendix 1 Definitions used for Time, Distance and Cost savings.

Multimedia Appendix 2 Volume of patients seen in the stroke prevention clinic during the pilot phase.

Multimedia Appendix 3 Distance and time savings for patients.

Multimedia Appendix 4 Ambulatory care costs for CACS codes (E751 and E752) in Ontario and Alberta for 2016/2017.

## Abbreviations

CACS: Comprehensive Ambulatory Classification System
CIHI: Canadian Institute for Health Information
eVisit: electronic visit
IQR: interquartile range
KHSC: Kingston Health Sciences Centre
MOHLTC: Ministry of Health and Long-Term Care
OOP: out-of-pocket
OTN: Ontario Telemedicine Network
SDM: substitute decision maker
