# Supplementary material for: Home Virtual Visits for Outpatient Follow-Up Stroke Care: Cross-Sectional Study
Source: J Med Internet Res. 2019 Oct 7;21(10):e13734. doi: 10.2196/13734 (PMC6803894; doi:10.2196/13734)
Supplement: Multimedia Appendix 1 [file jmir_v21i10e13734_app1.pdf]

Multimedia Appendix 1

Definitions used for Time, Distance and Cost savings

Travel distance	The two-way distance from the patient's home location to the hospital
Travel time	The time to commute from the patient's home to the hospital
In-person visit time	The time spent by the patient to find and pay for parking, time to walk from the parking lot into the hospital and to the clinic area and back, registration, and in-office waiting for the physician, etc. (excludes the actual time spent with the physician)
Total time	Travel time + in-person visit time
**Out-of-pocket expenses**	
Cost of travel	The cost of the trip, measured at 0.55 cents/km
Loss of pay	Hypothetical estimate of what an adult Canadian older than 25 years working fulltime would lose if they have visited the physician in the clinic for an in-person estimate assuming that they had similar travel distance and spent similar in-person time on average
Parking cost	Cost of parking in Kingston for 1 hour
Total out-of-pocket cost	Cost of travel + Loss of Pay + Parking cost
