# Supplementary material for: Home Virtual Visits for Outpatient Follow-Up Stroke Care: Cross-Sectional Study
Source: J Med Internet Res. 2019 Oct 7;21(10):e13734. doi: 10.2196/13734 (PMC6803894; doi:10.2196/13734)
Supplement: Multimedia Appendix 2 [file jmir_v21i10e13734_app2.pdf]

Multimedia Appendix-2

Volume of patients seen in the stroke prevention clinic during the pilot phase*

Total number of patients seen in the clinic (Aug 1 2018-Jan 31 219)	383
New patients seen*	194
Total Follow-up's (in-person and Evisit)	189
The proportion of E-visit Follow-up’s	40%
Evisits scheduled	81
Evisits completed	75
First-time Evisits	60
Follow up Evisits	15
Evisit no-show	2
Evisits canceled due to technical issues during test visit	2
Evisits canceled due to technical issues during the physician Evisit.	2

*The volumes reflect the patients seen by the one physician(RA) involved in the pilot study. The stroke prevention clinic is staffed by 3 physicians, and the workload is equally shared among them.
