# Supplementary material for: Home Virtual Visits for Outpatient Follow-Up Stroke Care: Cross-Sectional Study
Source: J Med Internet Res. 2019 Oct 7;21(10):e13734. doi: 10.2196/13734 (PMC6803894; doi:10.2196/13734)
Supplement: Multimedia Appendix 3 [file jmir_v21i10e13734_app3.pdf]

Multimedia Appendix - 3

Distance and time savings for patients

	Total	Mean (SD)	Median (IQR)
**Distance**			
Estimated travel distance avoided (Km)	4337.2	60.24 (67.12)	30.1 (11.2-82.2)
**Time**			
Estimated travel time saved (minutes)	3845.2	52.39 (42.25)	44 (21-69)
Estimated total time saved (minutes)	6005.2	88.55 (46.54)	80 (50-102)
Patient self-reported time saved (minutes)		85.37 (39.41)	75 (60-120)
