# Supplementary material for: Home Virtual Visits for Outpatient Follow-Up Stroke Care: Cross-Sectional Study
Source: J Med Internet Res. 2019 Oct 7;21(10):e13734. doi: 10.2196/13734 (PMC6803894; doi:10.2196/13734)
Supplement: Multimedia Appendix 4 [file jmir_v21i10e13734_app4.pdf]

Multimedia Appendix 4

Ambulatory care costs for CACS codes (E751 and E752) in Ontario and Alberta for 2016/2017

CACS code	Description	Median (CND)	Mean (CND)	
		Alberta	Alberta	Ontario
E751	General Signs / Symptoms / Examination /Investigations	$ 306.88	$ 334.04	$ 331
E752	Other Medical and Follow-up Care	$ 217.60	$ 304.33	$ 397
